# Good Timing Matters: The Spatially Fractionated High Dose Rate Boost Should Come First

**DOI:** 10.3390/cancers14235964

**Published:** 2022-12-02

**Authors:** Elisabeth Schültke, Felix Jaekel, Stefan Bartzsch, Elke Bräuer-Krisch, Herwig Requardt, Jean Albert Laissue, Hans Blattmann, Guido Hildebrandt

**Affiliations:** 1 Department of Radiooncology, Rostock University Medical Center, 18059 Rostock, Germany; 2 Department of Radiooncology, Technical University of Munich, 81675 Munich, Germany; 3 Institute for Radiation Medicine, Helmholtz Center Munich, 85764 Munich, Germany; 4 Biomedical Beamline ID 17, European Synchrotron Radiation Facility (ESRF), 38043 Grenoble, France; 5 University of Bern, 3012 Bern, Switzerland; 6 Independent Researcher, 5417 Untersiggenthal, Switzerland

**Keywords:** high dose rate radiotherapy, microbeam irradiation (MBI), pencilbeam irradiation (PBI), brain tissue tolerance

## Abstract

**Simple Summary:**

The administration of X-rays with therapeutic intent (radiotherapy) can cause severe unwanted adverse effects in tissues other than those that were the intended radiation target, such as tissues located in the path of the beam or close to the target region. The results of small animal studies suggest that the risk for adverse effects may be significantly reduced if the X-ray dose is administered extremely fast, as the so-called high dose rate radiotherapy. Microbeam irradiation and pencilbeam irradiation are two new experimental concepts of high dose rate radiotherapy with spatial dose fractionation at the micrometre range. The results of our studies show how the inclusion of these concepts into a conventional broad beam radiotherapy schedule could improve cancer radiotherapy for patients with malignant brain tumours.

**Abstract:**

Monoplanar microbeam irradiation (MBI) and pencilbeam irradiation (PBI) are two new concepts of high dose rate radiotherapy, combined with spatial dose fractionation at the micrometre range. In a small animal model, we have explored the concept of integrating MBI or PBI as a simultaneously integrated boost (SIB), either at the beginning or at the end of a conventional, low-dose rate schedule of 5x4 Gy broad beam (BB) whole brain radiotherapy (WBRT). MBI was administered as array of 50 µm wide, quasi-parallel microbeams. For PBI, the target was covered with an array of 50 µm × 50 µm pencilbeams. In both techniques, the centre-to-centre distance was 400 µm. To assure that the entire brain received a dose of at least 4 Gy in all irradiated animals, the peak doses were calculated based on the daily BB fraction to approximate the valley dose. The results of our study have shown that the sequence of the BB irradiation fractions and the microbeam SIB is important to limit the risk of acute adverse effects, including epileptic seizures and death. The microbeam SIB should be integrated early rather than late in the irradiation schedule.

## 1. Introduction

High dose rate radiotherapy is attracting increasing attention in the field of experimental radiotherapy. Observations that X-rays delivered at dose rates of ≥40 Gy/s cause only minimal adverse effects in the normal tissue environment and in organs at risk have been made in small animal models. This phenomenon, termed the FLASH effect [1,2], was observed in both brain [3,4,5] and lung tissue [1,6]. Data suggest that there is a differential response between the tumour and normal tissue and that ultra-fast dose deposition causes less inflammatory reaction in normal tissue than a comparable dose deposited at conventional dose rates [7,8]. In clinical broad beam radiotherapy, typical dose rates are 6–20 Gy/min. The FLASH effects have been achieved by working with electrons at modified clinical linear accelerators (LINACSs) [9,10] and with photons at synchrotron facilities [11].

FLASH radiotherapy usually designates a broad beam (BB) irradiation technique utilizing dose rates ≥ 40 Gy/s [1]. Taking high dose rate radiotherapy even a step further, two irradiation concepts are under development in which FLASH dose rates are combined with spatial dose fractionation at the micrometre range. Both monoplanar microbeam irradiation (MBI) and pencilbeam irradiation (PBI) have been developed at synchrotron beamlines dedicated to biomedical research. While the first manuscript highlighting the therapeutic potential of MBI was already published in 1998 [12], PBI has been explored primarily for its tissue-sparing effects [13,14].

In both monoplanar MBI and PBI, the normal brain tissue tolerance appears to be remarkably high [13,15] and the memory function appears to be well preserved [16,17]. Both MBI and PBI are characterized by an inhomogeneous dose distribution with periodically alternating high dose (peak dose) and low dose (valley dose) zones in the targeted tissue. The valley dose was defined as the dose between the paths of the microbeams, at a width of 350 µm where the individual width of each microbeam is 50 µm and the centre-to-centre spacing is 400 µm. In PBI, a much smaller tissue volume is directly traversed by the microbeams, compared to MBI with both equal microbeam width and centre-to-centre spacing [13]. In normal tissue, very few, if any cell bodies survive in the paths of the microbeams delivered at doses of several hundred Gy. Assuming comparable valley doses, PBI could be an approach to minimize the morphological damage and result in a better preservation of tissue function, at the same rate of tumour cell destruction as that seen with monoplanar MBI.

Similar to the broad beam FLASH, MBI affects the tumour tissue differently than normal tissue [18,19]. When focused on a macroscopic tumour, one single fraction of MBI, alone or included in a conventional radiotherapy schedule, can control the tumour much better than conventional radiotherapy alone [20,21]. When two fractions in a conventional radiotherapy schedule were replaced by MBI and the valley dose was equal to the conventional single fraction dose (orthovolt range) in a model of young adult Fisher rats bearing a highly malignant brain tumour (F98 glioma), a significantly increased recurrence-free survival interval and a significantly longer overall survival were achieved, compared to animals treated with conventional broad beam radiotherapy alone [21]. In that study, the peak dose acted as simultaneously integrated boost (SIB).

The inclusion of spatially fractionated high dose rate radiotherapy into a low dose rate radiotherapy schedule could be a suitable approach to increase the tumour response at clinically acceptable normal toxicity levels for patients with multiple brain metastases or multifocal glioblastoma multiforme. Following up on an earlier study where a monoplanar MBI SIB was included in a conventional, low dose rate whole brain radiotherapy (WBRT) protocol, we have now designed a study to also explore PBI as SIB in an otherwise low dose rate BB WBRT protocol. In a small animal model, we compared high grade acute normal tissue toxicity, specifically the occurrence of epileptic seizures and death, between animals irradiated in an exclusively low dose rate irradiation protocol, animals with a microbeam SIB included at either the beginning or the end of the low dose rate irradiation protocol and a control group of non-irradiated animals. The aim of this study was to assess the relevance of the timing for the microbeam SIB. Assuming that the tumour cell destruction in the irradiation target is caused by both direct damage as result of the ionizing irradiation (enhanced in the paths of the microbeams) and the bystander effects, an accompanying in vitro study using F98 glioma cells was conducted to assess the tumoricidal potential in irradiation schedules similar to those used in the small animal study.

## 2. Materials and Methods

### 2.1. Technical Setup Broad Beam Irradiation (Low Dose Rate)

Broad beam (BB) irradiation at a conventional low dose rate was conducted at ambient temperature (22.7 °C) using an X-ray Generator (Philips, Amsterdam, The Netherlands) located at the biomedical beamline ID 17 of the European Synchrotron Radiation Facility (ESRF) in France. The X-ray generator, working in the kV (orthovoltage) range, was operated with a 0.2 mm copper filter at an energy of 200 keV. Dose rates between 0.9245 and 0.942 Gy/min were measured in a water phantom at 1 cm depth. The duration to deliver the target dose of 4.0 Gy was between 4.26 and 4.32 min.

### 2.2. Technical Setup Synchrotron Irradiation (High Dose Rate)

The high dose rate irradiation experiments were conducted at the biomedical beamline ID 17 of the ESRF. The incident photon beam at ID 17 was modified by a wiggler set to its minimum gap of 24.8 mm, to benefit from the maximum available photon flux and passed through a set of Cu and Al filters. The spectrum used for the microbeam studies at this beamline is typically 50–350 keV, with a maximum intensity at approximately 105 keV [22].

Both the monoplanar microbeam irradiation (MBI) and pencilbeam irradiation (PBI) are microbeam irradiation techniques. As a basis for both, an array of quasi-parallel microbeams with an individual beam width of 50 µm spaced at a centre-to-centre distance of 400 µm was generated by inserting a fixed-space tungsten multislit collimator (UNT, Morbier, France) with an individual microbeam width of 50 µm, spaced at a 400 µm centre-to-centre distance into the incident beam [23]. In the irradiation target, this produces an inhomogeneous dose distribution characterized by a repetitive pattern of high (peak) dose zones and low (valley) dose zones. At the synchrotron, other than in clinical radiotherapy, the position of the synchrotron beam is fixed. To cover irradiation fields that are larger than the incident beam, the irradiation target needs to be moved through the beam. Since the maximum achievable synchrotron beam at the irradiation position was only a few millimetres high, the sample was moved vertically through the beam to cover the target. The dose deposition was regulated by modifying the beam height and the speed of the vertical movement through the synchrotron beam, while the multislit collimator was in a fixed position. A fast shutter system [24], positioned upstream from the multislit collimator and synchronized with the vertical translation of the goniometer, allowed a precise selection of the irradiation field and the pre-calculated speed under consideration of the decreasing machine current.

**Figure 1:**
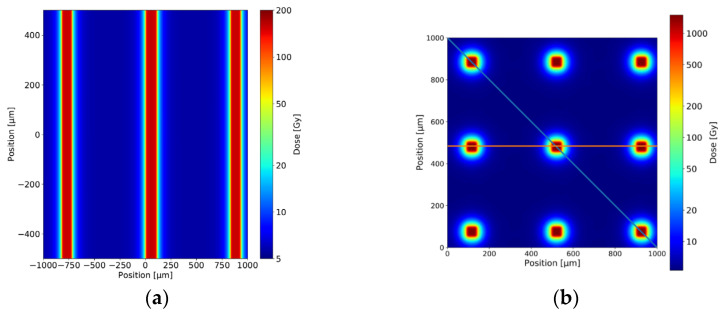
Scaled schematic of the dose distribution in a given target region seen in an upstream-to-downstream direction, shown for the monoplanar MBI with 50 µm wide microbeams (**a**) and PBI generated with the same multislit collimator used for the monoplanar MBI and additional horizontal fractionation, resulting in a grid of 50 µm × 50 µm pencilbeams (**b**). The centre-to-centre distance is 400 µm for both MBI and PBI.

For the monoplanar MBI, the irradiation target was moved vertically through the beam in a continuous movement at constant speed (19 mm/s). For PBI, in addition to the multislit collimator, the target was moved stepwise through a set of three 1200 µm high horizontal slits to generate a grid of 50 µm × 50 µm pencilbeams spaced laterally and vertically at a centre-to-centre distance of 400 µm (Figure 1).

A high peak-to-valley dose ratio (PVDR) is essential for a good normal tissue preservation. Thus, a fast dose deposition is required, in order to preserve a steep dose decrease at the microbeam edges and limit the dose blurring at the beam edges through a physiologic movement, such as a heartbeat or breathing. The dose rate in our study was measured by a semiflex ion chamber (PTW, Freiburg, Germany), scanning vertically through a 2 × 2 cm field at 2 cm depth in solid water, at a speed of 100 mm/s. At machine storage ring currents between 152 mA and 198 mA, dose rates of approximately 70 Gy/s/mA were achieved at the irradiation position.

### 2.3. Dose Calculation and Simulation

**Figure 2:**
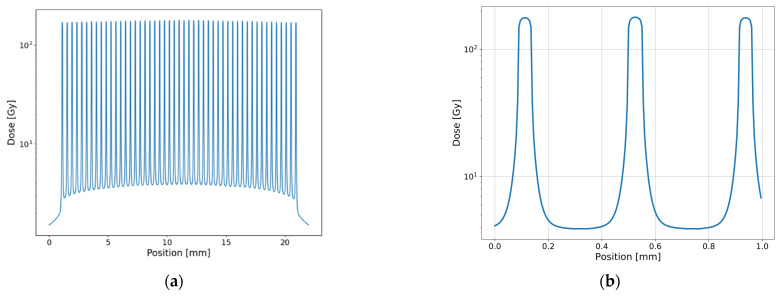

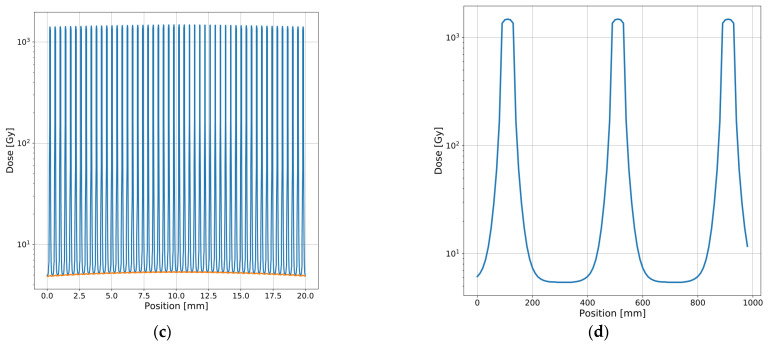
Results from the Monte Carlo simulation for MBI and PBI at a 1 mm depth in water (cell layer of in vitro exposures). Profiles of MBI with a peak dose of 174 Gy (**a**,**b**). Horizontal (**c**) and diagonal (**d**) profiles of PBI across the field @ 1500 Gy (compare Figure 1).

The dose distribution in the tissue and in the cell layer of the in vitro experiment (equivalent to 1 cm and 1 mm depth in water) was calculated using Monte Carlo simulations in the toolkit GEANT4 (version 10.4.2). The Livermore low energy physics libraries were used for these simulations, the range cut-offs for the electrons and photons were set to 1 µm. The simulations were performed in their semi-adjoint form [25] and the source model was adapted from [26]. The field sizes were 8.5 × 18 mm² for the small animal study (mouse) and 38 × 38 mm² for the in vitro experiment. The microbeams hit the water phantom of 40 mm thickness and the energy was scored at a mesh size of 1 × 1 × 0.005 mm (MBI) and 1 × 0.01 × 0.01 mm (PBI) with the highest resolution in the direction of the spatial fractionation. A total number of 10^9^ photons were simulated following the ESRF preclinical spectrum [22]. The collimator leakage with a harder X-ray spectrum was also taken into account [26]. Figure 2 shows the simulated microbeam dose profile for the in vitro exposures in the MBI and PBI techniques.

In the centre of the monoplanar MBI irradiation field, the maximum peak dose in the in vitro and in vivo experiments was 174 Gy. The maximum valley dose was 3.5 Gy and 4.4 Gy in the in vitro and in vivo exposures. In the centre of the PBI irradiation field, the maximum peak dose was 1500 µm and 1980 µm in the in vitro and in vivo experiments, respectively. The respective average valley dose, in the centre between four adjacent beams was 4.3 and 4.7 Gy. The valley doses varied substantially across the radiation field and were around 30% lower at the field edges.

The comparison between the irradiation techniques resulting in a highly inhomogeneous X-ray dose distribution, such as monoplanar microbeam irradiation and PBI with BB exposures, the concept of the equivalent uniform dose (EUD) was used, as originally defined by [27]. The EUD is the homogeneous dose that leads to the same cellular survival as an inhomogeneous dose distribution, assuming that the cells react independent of each other to the local dose they receive, according to the linear quadratic model (LQM). The LQM parameters α and β were assumed as 0.1 Gy^−1^ and 0.05 Gy^−2^ [28,29,30] and the EUD was retrieved by equating the homogeneous and inhomogeneous survival using

$$\bar{S}=exp\left(-\alpha \cdot EUD-\beta \cdot EUD^{2}\right)=\frac{1}{V}\int_{V}^{}d^{3}\vec{r}\;e^{-\alpha \cdot D\left(\vec{r}\right)-G\cdot \beta \cdot D^{2}\left(\vec{r}\right)}{}^{\;}.$$

For the in vivo MBI study, the EUD in a 1 cm depth (approximately the position of the brain) was 4.7 Gy and in the in vitro MBI exposures 6.0 Gy. The EQD2 of the entire course of the fractionated treatment for the BB only (5 × 4 Gy), the in vivo MBI SIB + BB (4 × 4 Gy + 6 Gy) and in vitro MRT SIB + BB (4 × 4 Gy + 4.7 Gy) was 30, 32 and 36 Gy.

For PBI, the EUD was 6.7 in vitro and 7.2 in vivo. The EQD2 of the entire fractionation schedule was 38.6 and 40.6 Gy for the in vivo and in vitro, respectively.

### 2.4. Small Animal Study

The experiments were conducted at the biomedical beamline ID17 of the ESRF in France (permit number 14ethax210 of the ESRF Ethics Committee, ETHAX 113, authorisation 28 May 2015).

Sixty young adult C57 BL/6J mice (Charles River, France) were used for this study. The animals were housed and cared for in a temperature-regulated animal facility exposed to a 12-h light/dark cycle. For all irradiation procedures, the animals were under general anaesthesia, induced by inhalation of 1.5–2% Isoflurane in compressed air and upheld by an intraperitoneal injection of a Ketamine and Xylazine cocktail (Ketamine 1 mg/10 g, Xylazine 0.1 mg/10 g). To assure a reproducible position, the anaesthetized mice were placed on a special positioning device in the prone position, with their front teeth hooked around a fixed wire. The animals were distributed into six experimental groups (n = 10/group, Table 1):

Group 1 received five single fractions of 4 Gy WBRT on five subsequent days. Group 2 received no irradiation at all and served as controls.

The animals in groups 4–6 received four fractions of 4 Gy WBRT and, in addition, a WBRT microbeam SIB in either the monoplanar MBI or PBI mode. SIB concepts are frequently used in clinical radiotherapy, to increase the biological efficacy and to shorten the overall treatment time to increase the patient’s quality of life. The peak doses were calculated based on the daily BB fraction dose of 4 Gy serving as the valley dose. Thus, all animals, except those of the control group, received 5 × 4 Gy delivered to the entire brain.

Group 3 and 4 received a WBRT SIB in the uniaxial MBI technique, either at the beginning (Group 3) or at the end (Group 4) of the irradiation schedule.

Group 5 and Group 6 received a WBRT SIB in the PBI technique, either at the beginning (Group 5) or at the end (Group 6) of the irradiation schedule.

The mice were positioned prone, on top of a 3-axis Kappa-type goniometer (Huber, Rimsting, Germany) with three prosilica cameras (Allied Vision Technologies GmbH, Stadtroda, Germany) supporting the reproducible positioning of each animal. The microbeam irradiation of the entire skull was performed by a vertical translation of the rat through the beam.

The conventional, low dose-rate irradiation in the broad beam technique was delivered from above, in the dorsal-to-ventral direction. The microbeam irradiation in the MBI and PBI techniques was conducted in the right-to-left lateral direction.

To assure that the entire brain was inside the irradiation target and to spare other tissue as much as possible, a 2D X-ray image was obtained prior to the high dose rate SIB, after which the target position was corrected, if necessary. The animals were sacrificed at 48 h and 7 days after the administration of the last irradiation fraction. The brains were carefully extracted from the skull, fixed in 10% phosphate-buffered formalin for 24 h and then stored in 1x PBS for later processing.

### 2.5. In Vitro Model

To assess the tumouricidal potential of the tested irradiation schedules in the glioma cells, we conducted an in vitro study using the commercially available F98 glioma cell line (CRL-2397, ATCC, USA, rodent origin). Due to this cell line’s characteristics, such as a high proliferation rate and invasive growth into normal brain structures, F98 glioma cells are frequently used to simulate the malignant human brain tumor glioblastoma multiforme [31]. F98 glioma cells are considered highly radioresistant and are therefore well suited to assess the therapeutic potential of new radiotherapy techniques. The cell line is well established in our laboratory for both in vitro and in vivo studies to follow up in vitro experiments with an in vivo study. The cells were cultivated in growth medium containing DMEM (31966-21, Gibco), 10% fetal bovine serum and 1% penicillin/streptomycin mixture, and harvested after aspirating the growth medium and incubating for approximately 20 min in a calcium- and magnesium-free medium, in a standard incubator.

The exponentially growing F98 cells were split into groups to match the irradiation conditions of the in vivo study.

### 2.6. Analysis of the Experimental Data

Cell proliferation: The F98 glioma cells were seeded in 30 mm diameter Petri dishes three days before the first irradiation, harvested and counted at 12 and 72 h after the last irradiation, using a hemocytometer. The cell numbers were plotted in the logarithmic mode using GraphPad Prism software.

Bystander effects in the tumour cell cultures: For this study, the growth medium of the irradiated cells was collected at 12 h after irradiation and added to non-irradiated glioma cell cultures. The work hypothesis was that the proliferation of the tumour cells which are not directly irradiated, nevertheless might be decreased when exposed to the growth medium which had been in contact with the irradiated cells. Prior to the irradiation, all growth medium was aspirated, leaving only a thin fluid film on the cultures during the irradiation. Immediately following the irradiation, fresh (non-irradiated) growth medium was added to all cultures. This medium was collected 12 h later. Then, 1 mL of this medium, which has been exposed to irradiated cells, was added to the non-irradiated cell cultures already submerged in fresh growth medium. In other words, the medium exposed to the irradiated cells was added to the naïve cells on top of, not instead of, the fresh growth medium. The cells were harvested and counted at 72 h after adding the medium exposed to the irradiated cells (bystander medium).

Clonogenic assay: Twenty-four hours prior to the first day of irradiation, 200 F98 glioma cells were seeded into T25 culture flasks, taking care to achieve a homogenous distribution of the single cells across the bottom of each flask. Thus, each viable cell could generate its own colony. These samples were submitted to the same irradiation schedules, as described for the in vivo study. Seven days after the last irradiation, the colonies were fixed with a 10% buffered formaldehyde solution and stained with 1% Cresyl violet. Each colony with a size of 50 cells or more was counted, assuming that each colony had arisen from one single glioma cell. The data were analyzed using the unpaired t-test (GraphPad Prism software).

Immunohistochemistry: The formalin-fixed and in paraffin embedded brains were sectioned 5.0 µm thick and mounted on microscope slides (SuperFrost^®^ Plus, R. Langenbrinck, Germany) for gamma H2AX immunostaining, as described previously [14]. Briefly, the tissue sections were deparaffinized and rehydrated by passing them through a series of alcohol and xylene washes, followed by vapour-based heat epitope retrieval in a citrate solution at pH6 (Target retrieval solution, Dako, Germany) at a temperature of 95 °C for 40 min. The tissue sections were then blocked with 100 μL of 1× PBS, 5% goat serum, and 0.3% triton X-100 buffer for 60 min at room temperature, followed by the incubation with gamma H2AX (Abcam 22551, Cambridge, UK), as the primary antibody at a dilution of 1:100 for 1 h at room temperature. Finally, the tissue sections were incubated with Alexa Fluor 488 at a dilution of 1:200 (Thermo Fisher Scientific, Waltham, MA, USA) as a secondary antibody and DAPI for 1 h at room temperature in the dark. Following the thorough rinsing with PBS, the slides were cover-slipped with Dako Fluorescent Mounting Medium (Dako North Amerika Inc., Carpinteria, CA, USA) and microphotographs were obtained using a fluorescence microscope (BZ-X, Keyence Deutschland GmbH, Neu-Isenburg, Germany) with a camera and computer link. For the immunofluorescence of the gamma H2AX stain, the excitation wavelength was 544 nm with an emission at 488 nm. The immunostaining utilizes antibodies against the histone 139, which is only accessible after DNA-double-strand-breaks, such as those developing after irradiation. We have shown previously that the gamma H2AX antibody with a DAPI nuclear counterstain is reliable for the assessment of the DNA damage after MRT [14].

### 2.7. Statistic Analysis

A non-parametric One-Way ANOVA test (GraphPad Prism 6, GraphPad Software, Inc., La Jolla, CA, USA). was used to assess the statistical significance of the data in the in vitro study (cell numbers and colony counts).

## 3. Results

The results of this study suggest a peak dose-dependent normal tissue toxicity for microbeam radiotherapy. Furthermore, this toxicity is dependent on the sequence of low dose rate BB and high dose rate microbeam irradiation fractions.

### 3.1. Health Status, Neurologic Signs and Acute Adverse Effects In Vivo

In the animals receiving either 5 × 4 Gy low dose rate irradiation only or low dose rate irradiation combined with an either early or late SIB of monoplanar MBI with peak doses of 174 Gy, only one adverse effect occurred: starting with the third day, the animals required more anaesthetic to be reliably positioned during irradiation. No signs of increased brain pressure, such as circling or inactivity, were observed.

In the animals receiving a PBI SIB with peak doses of 1980 Gy, at the end of the low dose rate radiotherapy schedule, four out of 10 animals died within 2 h after irradiation. Two of the animals were observed to have a generalized epileptic seizure and stopped breathing afterwards. All animals had woken up from anaesthesia, walked around their cage and started eating before, so an anaesthesia mishap can be excluded. No high grade acute adverse effects were observed in the animals which received the PBI SIB at the beginning of the low dose radiotherapy schedule.

The valley dose was approximately 4 Gy in both the monoplanar MBI and in the PBI schedules, in order to have all animals receive 4 Gy as a WBRT dose on all treatment days, with the microbeam peak doses acting as SIB. Therefore, the PBI peak dose must have been detrimental. Even higher PBI peak doses had been administered in the same mouse strain and with the same microbeam width and spacing in WBRT before without causing acute adverse effects; however, this had been carried out on healthy, not-pre-irradiated mice [13]. The fact that no adverse effects were seen in the animals which received the PBI SIB on the first irradiation day agrees with those findings. In the pre-irradiated animals, however, the low dose rate irradiation had already caused vascular damage, resulting in cerebral edema. In this setting, the radiosurgical high dose PBI beams caused an acute increase of vascular damage, followed by increased intracranial pressure sufficient to cause generalized seizures and subsequent death.

**Figure 3:**
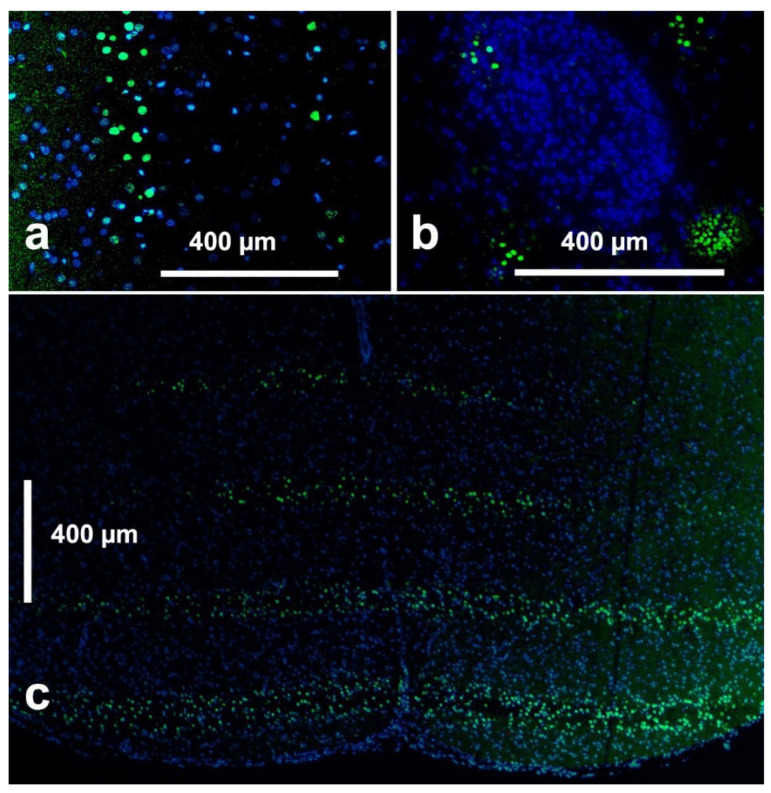
Immunostaining (gammaH2AX) highlighting irradiation-induced DNA double strand breaks. Lateral view after the monoplanar MBI (**a**), PBI (**b**) and the axial view (along the direction of the beams into the tissue): seen from above, it looks the same after the monoplanar MBI and PBI (**c**). Images shown are obtained from animals receiving the SIB first, to demonstrate the DNA double strand breaks corresponding to the beam geometry.

The beam geometry is reflected in the microphotographs of the immunostained brain tissue (Figure 3).

### 3.2. Tumour Cell Destruction In Vitro

**Figure 4:**
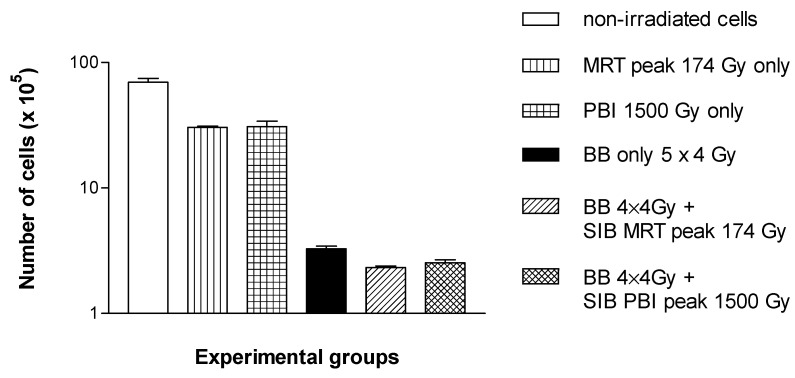
Cell counts after the late microbeam SIB. A significantly increased cell destruction, compared to the non-irradiated controls, was seen in all irradiated groups. The differences in the cell destruction were also statistically significant between the low dose rate and the high dose rate irradiation groups but not between the two high dose rate irradiation groups.

Similar to the results of the in vivo study, the results of the in vitro study also suggest that a spatially fractionated high dose rate SIB should be included into a low dose-rate schedule early rather than late. The cell proliferation assay conducted with cells receiving the high dose rate SIB on the last day of irradiation shows that cell death occurs continuously during the first 72 h after irradiation (Figure 4). While the number of non-irradiated control cells increases in this period, the number of irradiated cells decreases. While there is no significant difference between both of the high dose rate irradiation techniques, the difference between the low dose rate irradiation only and the low dose rate irradiation plus the high dose rate SIB is significant at 72 h after irradiation (*p* < 0.0001).

**Figure 5:**
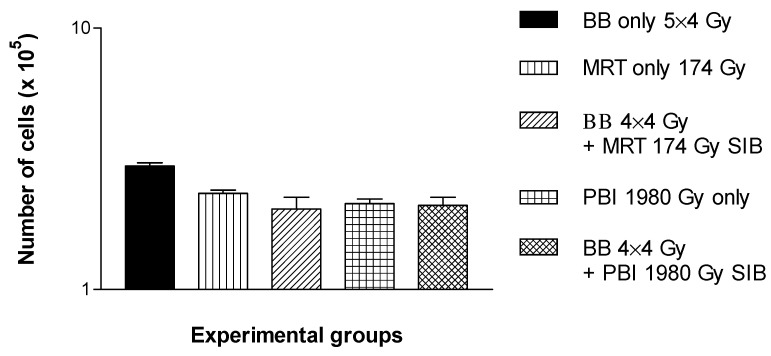
Secondary cell counts (with conditioned medium). Single fraction high dose rate, as well as high dose rate SIBs are significantly more effective than low dose rates only. There was no significant difference between the high dose rate techniques.

The addition of the bystander medium (exposed for 12 h to the irradiated cells, conditioned medium) to the non-irradiated tumour cell cultures again shows a significant difference between the low dose rate and the high dose rate techniques but none between the individual high dose rate techniques (Figure 5).

Based on the data shown and considering a significantly higher risk of death associated with PBI, a monoplanar MRT SIB seems to be the preferable one of the two tested spatially fractionated high dose rate irradiation techniques for inclusion as SIB into a low dose rate radiotherapy schedule.

**Figure 6:**
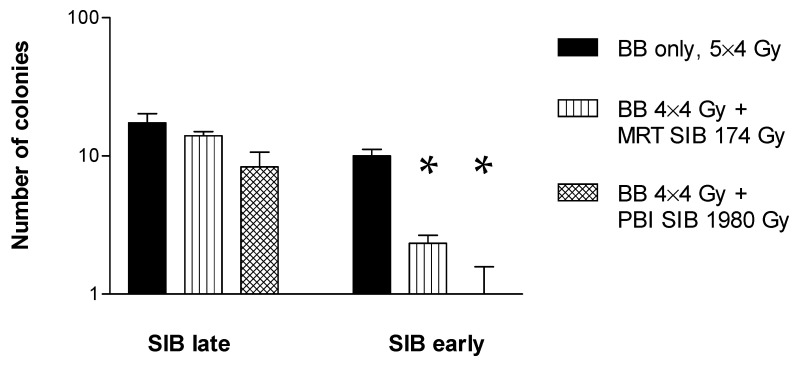
Colony counts after low dose rate irradiation with and without SIB. Only a small additional decrease in the number of colony counts was seen after the late SIB, but a highly significant additional decrease was achieved with an early spatially fractionated high dose rate SIB, compared to low the dose rate irradiation alone. The difference between the monoplanar MRT SIB and PBI SIB was also statistically significant. The cells were irradiated in the flasks directly. Error bars represent SEM. Asterisks are used to highlight statistically significant differences compared to the other two groups in the early SIB experiment.

The colony formation assay shows the same trend for the late microbeam SIB. However, for the early SIB, it shows a trend towards a more pronounced tumour cell inhibition after PBI, compared to the monoplanar MBI (Figure 6). In the samples from the two groups in which the high dose rate SIBs were administered early in the radiotherapy schedule, highly significant differences are seen between each of the three irradiation schedules (low dose rate only, MBI SIB + low dose rate and PBI SIB plus low dose rate).

## 4. Discussion

High dose rate irradiation techniques with photons are almost exclusively developed at synchrotron facilities. Thus, access is currently limited by the competition for experimental time. However, efforts to construct synchrotron-independent compact sources to produce the necessary photon flux are under way [32,33]. At this stage, high dose rate radiotherapy promises an extremely good preservation of the normal tissue function for human patients, even at single fraction doses far higher than those typically used in conventional radiotherapy. While BB FLASH radiotherapy can technically be delivered in unlimited numbers of subsequent fractions, spatially dose-fractionated techniques such as MBI and PBI are limited to one single fraction: A precise repositioning with the micrometre precision required for irradiation on subsequent days is technically impossible in human patients, at least with currently available techniques. As a consequence, an exact dose prescription in the target zone would be possible only for one single MBI or PBI SIB fraction.

SIB concepts have gained popularity in conventional radiotherapy because they increase the biologically effective dose and thus increase the tumour control [34,35]. They shorten the radiotherapy schedule, which improves the quality of life for the patient. An improvement of the tumour control is desirable for both patients with multiple brain metastases and for patients with multifocal glioblastoma multiforme. The interval for the tumour recurrence after a course of conventional radiotherapy is, on average, less than a year for both tumour entities. While WBRT is accepted as a therapeutic concept for patients with multiple brain metastases, it is rejected on the grounds of a high risk for neurological adverse effects in patients with malignant primary brain tumours. In a typical 14 × 2.5 Gy course of WBRT for patients with multiple brain metastases, the BED would be lower than in a 13 × 3 Gy course for glioblastoma extended to the entire brain (43,75 vs. 50.7). However, with increasing survival times of patients with multiple brain metastases, due to improved systemic therapy, the risk for neurological deficits as late adverse effects also increases. High dose rate radiotherapy generally preserves the normal tissue function far better than low dose rate radiotherapy [1]. Further, high dose rate irradiation destroys tumour cells equally or even better than does low dose rate irradiation. Therefore, a high dose rate SIB with a spatially fractionated irradiation technique may also yield an improved tumour control and a limited risk of neurological deficits.

The comparison of the X-ray doses delivered in a microbeam geometry with conventional broad beam doses is challenging and extremely complex. Characteristic parameters of microbeam irradiation, such as a high peak dose, dose delivery in one single irradiation session and the inhomogeneous dose distribution with periodically alternating high dose (peak dose) and low dose (valley dose) zones in the target tissue, are hard to match with the parameters of a uniform, seamless broad beam irradiation, administered in a temporally fractionated series of normofractionated low dose rate radiotherapy. To solve this problem, a larger amount of quantitative biological data should be generated in preclinical microbeam studies, to be compared to the biological responses seen in matching the clinically fractionated irradiation schedules, both testing the same biological system. We are not aware of publications listing detailed results of such experiments.

FLASH radiotherapy with electrons at a modified clinical LINAC and the monoplanar MBI at the synchrotron have both already advanced to the stage of veterinary studies [36,37,38]. It remains questionable whether the timely sequence of the conventional irradiation and high dose rate radiotherapy boost is of any consequence. The data of an earlier study suggested that it might be wise to include a MBI SIB into a conventional WBRT schedule early rather than late, based on the better tumoricidal effect seen in an accompanying in vitro study [39]. The results of the current study strongly support this recommendation, from the aspects of both tumor cell destruction and of patient safety. Although the statistical power might be limited by the number of animals in each experimental group, the agreement with our earlier study reporting on the inclusion of a MBI SIB [39] in favour of including the SIB at the beginning rather than at the end of a conventional, low dose rate radiotherapy schedule, is encouraging.

While no adverse effects were observed in the MBI study, the radiogenic lethality was 40% after PBI with a comparable valley dose when the PBI SIB was administered at the end of the conventional radiotherapy schedule. However, no fatalities occurred with the PBI SIB as the first irradiation fraction. The death of the animals receiving a PBI SIB at the end of the radiotherapy schedule was surprising: considerably higher in-beam PBI doses had been administered—without dramatic side effects—by a WBRT concept in an earlier study [13] The reason for the death of the animals in the present study is most likely a more pronounced acute increase of intracranial pressure, due to intracerebral edema within the first two hours after the PBI SIB, in animals which had been pre-irradiated with conventional BB WBRT. This would fit the clinical picture of the observed epileptic seizures in the animals immediately before death. The damage to the tumour-supplying blood vessels (neovasculature) and the development of vasogenic cerebral edema after WBRT has been described [40]. Thus, the advantages of a high dose rate SIB, scheduled early rather than late, consist in a lower risk of death and in a significantly higher percentage of cell destruction, compared to the low dose rate radiotherapy. To take advantage of this, it might be worth considering the inclusion of an MBI SIB into a prophylactic clinical low dose rate WBRT schedule. With a valley dose of 2 Gy, which is typical for prophylactic WBRT, the toxicity would be significantly lower than in our study. The resulting MRT peak dose would also by significantly lower than in our study, below 100 Gy. However, considering the increase of the biological effective dose achievable and seeing the therapeutic success with comparably low peak doses in the veterinary MRT study currently conducted at the ESRF, it might be worthwhile to test such a concept.

## 5. Conclusions

The results of this study support the work hypothesis that the integration of a high dose rate microbeam SIB into a conventional, low dose rate schedule of whole brain radiotherapy, increases the tumour cell destruction without producing inacceptable acute adverse effects if the boost is administered at the beginning of the radiotherapy schedule. The sequence of conventional radiotherapy and high dose rate boost in a spatially fractionated technique is highly important for the outcome, not only for a better tumour control but also as an aspect of patient safety. Given equal integrated doses, the normal tissue toxicity increases with increasing doses in the paths of the microbeams. Compared to PBI, the monoplanar microbeam irradiation (MBI) appears as the safer option for a SIB integrated in a conventional BB WBRT schedule. When incorporated into a conventional, low dose rate BB WBRT schedule in the scenario described in this study, the microbeam SIB should be administered early, rather than late.

## Figures and Tables

**Table 1:** Overview over the experimental groups and the treatment administered. BB: low dose rate broad beam irradiation; MBI: monoplanar microbeam irradiation; PBI: pencilbeam irradiation; SIB: simultaneously integrated boost.

	Day 1	Day 2	Day 3	Day 4	Day 5
**Group 1**	4 Gy BB	4 Gy BB	4 Gy BB	4 Gy BB	4 Gy BB
**Group 2**	------------------------- Control, no irradiation ---------------------------				
**Group 3**	MBI SIB	4 Gy BB	4 Gy BB	4 Gy BB	4 Gy BB
**Group 4**	4 Gy BB	4 Gy BB	4 Gy BB	4 Gy BB	MBI SIB
**Group 5**	PBI SIB	4 Gy BB	4 Gy BB	4 Gy BB	4 Gy BB
**Group 6**	4 Gy BB	4 Gy BB	4 Gy BB	4 Gy BB	PBI SIB

## Data Availability

Data are available upon request from the primary investigator (E.S.)
